# Prototype-based contrastive substructure identification for molecular property prediction

**DOI:** 10.1093/bib/bbae565

**Published:** 2024-11-04

**Authors:** Gaoqi He, Shun Liu, Zhuoran Liu, Changbo Wang, Kai Zhang, Honglin Li

**Affiliations:** School of Computer Science and Technology, East China Normal University, 200062 Shanghai, China; School of Computer Science and Technology, East China Normal University, 200062 Shanghai, China; School of Computer Science and Technology, East China Normal University, 200062 Shanghai, China; School of Computer Science and Technology, East China Normal University, 200062 Shanghai, China; School of Computer Science and Technology, East China Normal University, 200062 Shanghai, China; Innovation Center for AI and Drug Discovery, East China Normal University, 200062 Shanghai, China; Shanghai Key Laboratory of New Drug Design, School of Pharmacy, East China University of Science & Technology, 200237 Shanghai, China

**Keywords:** molecular property prediction, Graph Neural Networks, self-supervised learning, contrastive learning

## Abstract

Substructure-based representation learning has emerged as a powerful approach to featurize complex attributed graphs, with promising results in molecular property prediction (MPP). However, existing MPP methods mainly rely on manually defined rules to extract substructures. It remains an open challenge to adaptively identify meaningful substructures from numerous molecular graphs to accommodate MPP tasks. To this end, this paper proposes **P**rototype-based c**O**ntrastive **S**ubstructure **I**dentifica**T**ion (POSIT), a self-supervised framework to autonomously discover substructural prototypes across graphs so as to guide end-to-end molecular fragmentation. During pre-training, POSIT emphasizes two key aspects of substructure identification: firstly, it imposes a soft connectivity constraint to encourage the generation of topologically meaningful substructures; secondly, it aligns resultant substructures with derived prototypes through a prototype-substructure contrastive clustering objective, ensuring attribute-based similarity within clusters. In the fine-tuning stage, a cross-scale attention mechanism is designed to integrate substructure-level information to enhance molecular representations. The effectiveness of the POSIT framework is demonstrated by experimental results from diverse real-world datasets, covering both classification and regression tasks. Moreover, visualization analysis validates the consistency of chemical priors with identified substructures. The source code is publicly available at https://github.com/VRPharmer/POSIT.

## Introduction

Molecular property prediction (MPP) is a significant task in modern drug discovery. Accurate prediction methods can accelerate lead compound discovery, virtual screening, and other drug discovery processes [1, 2]. However, conventional wet-lab experiments entail considerable time and labor costs [3]. In recent years, deep learning techniques have been widely applied in various downstream tasks and have reported excellent results. With the continuous accumulation of biochemical data, deep learning-based MPP methods are attracting an ever-increasing interest from researchers, showing potential in prediction performance and generalization capability [4].

Deep learning-based MPP methods can be divided into sequence-based and graph-based methods according to the representation of molecules [5]. Each presents unique advantages and challenges for predicting molecular properties. Sequence-based methods mainly leverage the Simplified Molecular Input Line Entry System (SMILES) strings [6], which can benefit from advanced language models [7, 8]. However, the string representations loss spatial structural information [9, 10]. In contrast, graph-based methods offer a topology-aware representation by viewing atoms as nodes and bonds as edges. Consequently, Graph Neural Networks (GNNs) have become well-suited tools for MPP tasks. GNN-based methods continue to emerge and achieve promising results [1, 11].

Existing enhancements to graph-based MPP methods are primarily driven by the unique characteristics of molecules. Typical contributions include D-MPNN [12], which fuses edge feartures to the message passing phase for incorporating attributes of chemical bonds. Attentive FP introduces a multi-level attention mechanism to enable both reasoning capabilities and interpretability [13]. Combining chemical domain knowledge, FP-GNN integrates fingerpints into the graph representations of molecules [14]. These GNN-based models mainly consider atom-level feature aggregation, while the rich structural information of functional groups remains to be explored. According to the chemical domain knowledge, functional groups (substructures) form the basic building blocks that determine molecular properties and are shared across molecules [15, 16]. For example, the carboxyl group (-COOH) often indicate compounds with high water solubility.

To this end, recent MPP-related works have considered substructures in the learning process. These methods mainly adopt manually defined fragmentation rules. For instance, FraGAT randomly breaks acyclic single bonds to generate substructures [17]; HiGNN employs the Breaking of Retrosynthetically Interesting Chemical Substructures algorithm to partition molecules [18]; MgRX combines the BRICS and the Retrosynthetic Combinatorial Analysis Procedure algorithm to obtain fine-grained fragments [19]; and CAFE-MPP detects breakable bonds for fragmentation according to chemistry-aware rules [20].

**Figure 1:**
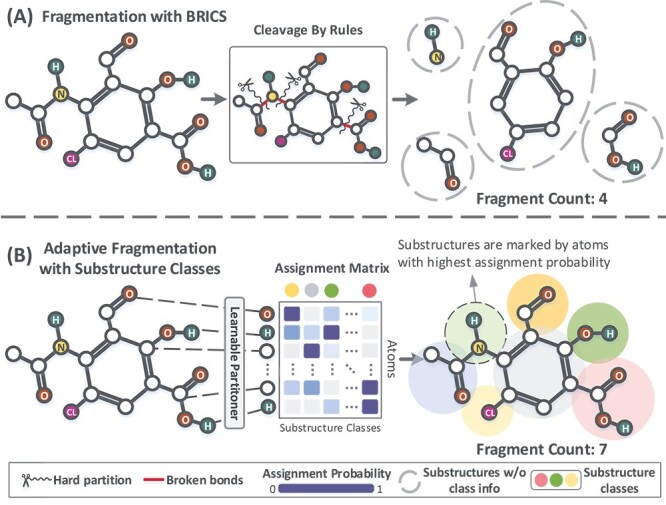
(A) Rule-based molecular fragmentation. Using the BRICS algorithm, the molecule is fragmented into four substructures by pre-defined rules. Each substructure does not possess a global substructure class information. (B) Adaptive fragmentation with POSIT. The molecule is divided into seven substructures by probabilistically assigning each atom to a global set of substructure classes. The partitioning is implemented by imposing both topology-based and attribute-based constraints simultaneously.

These rule-based methods leverage chemical knowledge to cleave molecules, but they lack flexibility and are not aware of substructure classifications. For example, Fig. 1(A) demonstrates that both halogen (Cl) and phenolic hydroxyl, which are different classes of functional groups. This mixture obscures the functional groups that may be important for prediction.

As for adaptive substructure identification methods, though not directly designed for MPP tasks, there are two related directions: graph mining and graph clustering. Graph mining aims at the data-driven discovery of frequent subgraphs, which may demand extensive domain knowledge and high computational costs [21, 22]. Notable methods for graph clustering, such as DiffPool and MinCutPool [23, 24], hierarchically partitions nodes into different clusters and generates pooled representations. Moreover, MICRO forms various motifs via node clustering with the EM algorithm [25]. SLIM models structural interaction through mapping rooted subgraphs to finite landmarks, while flexibly shaped substructures cannot be discovered [26]. These works primarily utilize node-level similarities to cluster nodes on each graph. However, substructure-level relationships across graphs are not fully exploited to model the intra-class consistency and inter-class discrimination of substructures.

To address the above concerns, we propose **P**rototype-based c**O**ntrastive **S**ubstructure **I**dentifica**T**ion (POSIT) framework to adaptively mine substructures, consequently augmenting molecular representations for MPP tasks. Compared to existing rule-based methods, POSIT has several merits: (1) it allows fine-tuning the fragmentation process based on downstream supervised signals, leading to more informative substructure representations; (2) it results in flexible, class-aware substructures with global coherence; and (3) it eliminates the reliance on chemistry domain knowledge. The framework incorporates two learning stages. During pre-training, a graph encoder and a partitioner are pre-trained to partition molecules. Specifically, the partitioner softly assign nodes to various substructure classes with a connectivity constraint. On this basis, a prototypical contrastive objective is designed on substructure-level representations, thereby encouraging salient clustering of substructures with intra-class consistency and inter-class discrimination.

In the fine-tuning stage, the predictor is trained via supervised MPP data, where a cross-scale attention mechanism is introduced to capture the interaction between the the substructure-level and the graph-level representations. Meanwhile, the encoder and the partitioner are fine-tuned for downstream MPP tasks. We conducted extensive experiments on 10 datasets to validate the performance of POSIT, covering classification and regression tasks.

The contributions of this work are summarized as follows:

- We introduce an innovative self-supervised framework capable of adaptively extracting informative substructural prototypes among biochemical data, consequently identifying meaningful molecular substructures. Further visualization studies illustrate the consistency of the partitioned substructures with chemical priors.
- The prototypical contrastive substructure identification is explored as a novel pretext task for further fine-tuning. During fine-tuning, a cross-scale attention mechanism is integrated, which fuses substructure-level information to enhance molecular representations.
- Comprehensive experiments are used to evaluate the performance of POSIT, covering classification and regression MPP tasks on 10 real-world datasets. Results compared to baseline models and ablation studies demonstrate the effectiveness and generalizability of POSIT.

## Materials and methods

In this section, we first state the problem definition of MPP and then introduce the preliminary requirements of molecular fragmentation. Subsequently, we elaborate on the design of the two-stage framework, emphasizing both pre-training and fine-tuning stages.

### Problem definition

Typically, a molecule can be viewed as an undirected graph $G=\langle V,E \rangle $, where $V$ denotes the node set representing atoms and $E\subseteq V\times V$ denotes the edge set representing chemical bonds. The initial features of $n$ nodes within a graph are represented as $\mathbf{X} \in \mathbb{R}^{n \times d_{1}}$, where $d_{1}$ is the dimension. Connection relations are represented as an adjacency matrix $\mathbf{A} \in \mathbb{R}^{n \times n}$ and edge features $\epsilon \in \mathbb{R}^{|E| \times d_{2}}$, where each element of $\mathbf{A}$ is either 0 or 1. Given a set of $N$ molecular graphs: $\{G_{1},G_{2},\ldots ,G_{N}\}$, and their task labels on various properties: $\{y_{1},y_{2},\ldots ,y_{N}\}$, the objective of MPP is to train a model $F$, where $\widehat{y}=F(G)$ fits various ground truths well. This model requires informative molecular representations that applicable across varied tasks.

### Substructure identification

Matching exact functional groups in molecules is related to graph mining, which may require expensive computational costs [22]. Hence, we expect to discover substructures that potentially encapsulate functional groups. During this process, the challenge lies in designing a method to partition atoms into coherent and meaningful substructures that benefit downstream tasks.

Following the requirements of graph mining, substructures should adhere to certain constraints [22, 27]: (1) Connectivity: since functional groups are composed of local atomic clusters, substructures should maintain tight connectivity. (2) Topological similarity: substructures within the same class should represent similar connectivity patterns. (3) Attribute consistency: as for attributed graphs, substructures within the same class should share identical type and count of nodes. To meet the challenges, we considers to relaxing these constraints to probabilistic versions, thereby enabling learnable fragmentation in a data driven manner. Details are described in the following subsections.

### Overview

**Figure 2:**
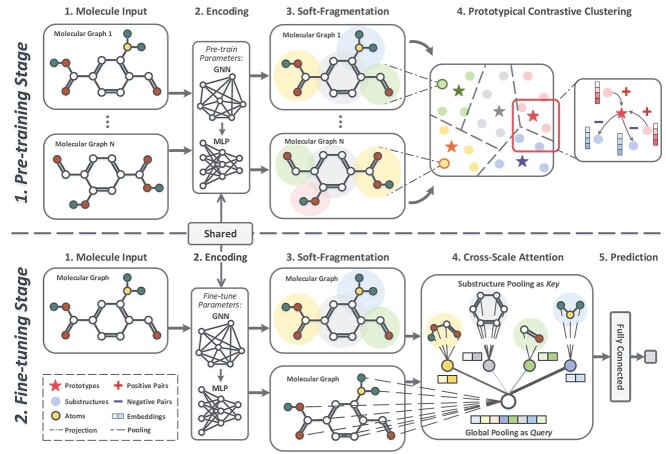
The overall architecture of POSIT. (1) **Stage-I:pre-training stage.** Encoded molecular atoms are softly partitioned into substructures. The prototypical contrastive objective directs these substructures to cluster by pulling them closer to their own class prototypes and distancing them from others. (2) **Stage-II: fine-tuning stage.** Substructures are first identified by the pre-trained network. Next, the cross-scale attention fuses the substructure-level information with the global representation, which is finally fed into the predictor.

The overall architecture of the two-stage network is illustrated in Fig. 2. The pre-training stage aims at adaptively identifying meaningful substructures. Specifically, a GNN-based encoder and a partitioner are jointly pre-trained under two objectives: (1) a local connectivity constraint that generates geometrically meaningful subgraphs within a molecule; and (2) a global prototype-based contrastive clustering loss that encourages substructures form salient clusters, using both topological and attribute-based similarities.

The fine-tuning stage leverages the supervised data to update pre-training parameters and build accurate predictors for MPP tasks. In particular, based on the identified substructures, the cross-scale attention mechanism is introduced to integrate substructure-level representations into graph-level representations as informative features.

### Pre-training stage

Given a molecular graph $G$ with $n$ nodes, we first extract $d$-dimensional node embeddings with a GNN-based encoder. Utilizing message passing, the representation of node $i$ at the $l$th layer is aggregated iteratively from its neighbour set $N(i)$:


(1) \begin{align*}& \mathbf{h}_{i}^{(l+1)}=\text{Upd}\left(\mathbf{h}_{i}^{l},\text{Agg}(\{\mathbf{h}_{j}^{l},\forall j \in N(i)\})\right)\end{align*}

where $\text{Agg}(\cdot )$ is the aggregation function, and $\text{Upd}(\cdot )$ is the updating function. Any variant of GNNs is applicable [28]. Here, Attentive FP [13], an attention-based graph encoder, is used in the implementation of POSIT.

Next, we aim at partitioning molecules into various substructures. Instead of directly specifying the number of substructures within each molecule, $K$ classes (or clusters) of substructures are defined globally across all molecules. Consequently, nodes within a molecule that are partitioned into the same class will naturally form a substructure. Given a molecular graph $G$ with $n$ nodes, we assign the embeddings of these nodes into $K$ classes via an MLP-based partitioner:


(2) \begin{align*}& \mathbf{h}^{\prime}_{i}=\text{MLP}(\mathbf{h}_{i}),\end{align*}

where $\mathbf{h}^{\prime}_{i}\in \mathbb{R}^{K}$, and $K$ denotes the pre-defined number of substructure classes. Next, the probability of node $i$ belonging to a certain substructure class $\mathbf{s}_{i}\in \mathbb{R}^{K}$ can be calculated as follows:


(3) \begin{align*}& \mathbf{s}_{i}=\text{Softmax}(\mathbf{h}^{\prime}_{i},\tau_{1})=\frac{\exp\left(\mathbf{h}^{\prime}_{i}\right)/\tau_{1}}{\sum_{j=1}^{K} \exp\left(\mathbf{h}^{\prime}_{i}[j]\right)/\tau_{1}},\end{align*}

where $\tau _{1}$ is the temperature parameter that controls the peakedness of the Softmax function. Hence, the node assignment matrix of $G$ is described as $S \in \mathbb{R}^{n\times K}$:


(4) \begin{eqnarray*} \mathbf{S}=[\mathbf{s}_{1}, \mathbf{s}_{2}, \ldots, \mathbf{s}_{n}]^\top, \end{eqnarray*}

which assigns each of the $n$ nodes into $K$ substructure classes.

#### Connectivity constraint

After partitioning molecular nodes into substructures through the partitioner, this subsection focuses on applying specific constraints to ensure that the resultant substructures should be geometrically meaningful. Generally, atoms in the same functional group are tightly connected, while connections between different functional groups are relatively sparse [16]. Inspired by this observation, a modularity-based regularization is introduced as a constraint, which is a spectral clustering metric that describes the connectivity of graph partitions. This constraint enforces that the assignment matrix $\mathbf{S}$ should create geometrically connected substructures. Here, we employed the relaxed version of modularity [29], which is formulated as follows:


(5) \begin{align*}& \mathcal{L}_{m}=\frac{1}{2m} \text{Tr} \left( \mathbf{S}^\top (\mathbf{A}-\frac{\mathbf{d}\mathbf{d}^\top}{2m}) \mathbf{S} \right),\end{align*}

where $\mathbf{d} \in \mathbb{R}^{n}$ is the degrees of nodes, $\mathbf{A} \in \mathbb{R}^{n \times n}$ is the adjancency matrix of the input graph, and $m=|E|$ denotes the edge count in the molecular graph $G$.

In practice, optimizing this objective tends to assign all nodes to a single partition [24]. To avoid such degenerate solutions, the orthogonality regularization is additionally included [30]. The formula is


(6) \begin{align*}& \mathcal{L}_{o}=\frac{\sqrt{K}}{n}\Big\vert\Big\vert \sum_{i=1}^{n}{\mathbf{s}_{i}} \Big\vert\Big\vert_{\mathrm{F}}-1.\end{align*}

This objective counts the size of substructure classes as regularization, reaching 0 when the sizes of different classes are strictly balanced.

Therefore, the overall loss function for connectivity is formulated as follows:


(7) \begin{align*}& \mathcal{L}_{con}=\mathcal{L}_{m}+\alpha\cdot\mathcal{L}_{o},\end{align*}

where $\alpha $ controls the balance of the two loss terms.

#### Prototypical contrastive substructure clustering

Besides tight connectivity, substructures of the same class are expected to share topological similarity and attribute consistency, which is identical with the clustering property. To explicitly enforce clustering among the substructure instances, we introduce the concept of prototypes.

Prototypes are the representative embeddings of classes, i.e. the centroids of classes [31, 32]. Leveraging prototypes to represent classes allows capturing intra-class similarity and inter-class dis-similarity between classes [33, 34]. In this work, prototypes are defined as representative substructures that guide substructures to form respective clusters. Concretely, POSIT minimizes the distance between substructure embeddings and their assigned prototypes, while maximizes the distance between substructure embeddings and all other prototypes.

First, substructure embeddings are derived. The probability of each node $i$ being assigned to substructure classes is indicated by the probability vector $\mathbf{s}_{i}$ from Equation (3). Therefore, the substructure embeddings in $G$ can be obtained by performing average pooling of the nodes inside each substructure. Let $\mathbf{S}$ be the node assignment matrix of $G$ as in Equation (4), the substructure embeddings in $G$ can then be computed as follows:


(8) \begin{align*}& \mathbf{Z} = \mathbf{S}^\top \mathbf{X} \oslash \left( \text{rep}\left(\sum_{j=1}^{n}{\mathbf{S}^\top_{[:,j]}},d\right) \right),\end{align*}

where $\mathbf{Z}\in \mathbb{R}^{K\times d}$ is the embedding matrix of substructures in $G$, $\mathbf{X} \in \mathbb{R}^{n \times d}$ is the embedding matrix of nodes in graph $G$, $\mathbf{S}^\top _{[:,j]}$ is a $K$ dimension vector representing the probability that the $j$th node is assigned to the $K$ substructure classes, $\oslash $ is the element-wise division operator, and $\text{rep}$ expands a $K$-dimensional vector to a ${K\times d}$ matrix. Each graph contains $K$ substructure embeddings in $\mathbf{Z}$ with dimension $d$, representing the occurrences of the $K$ substructure classes within this graph. Substructure classes that do not appear in $G$ are naturally represented as zero vectors in $\mathbf{Z}$. Therefore, the embeddings of the substructures that emerge in $G$ can be conveniently represented as the substructure embedding matrix $\mathbf{Z} = [\mathbf{z}_{1}, \mathbf{z}_{2}, \ldots , \mathbf{z}_{K}]^\top $. Subsequently, substructure prototypes will be derived from these classes of substructure embeddings across the dataset, with the count also equal to $K$.

**Substructure-prototype constrastive clustering.** After obtained the substructure embeddings, we employ a prototype-based contrastive clustering to enforce that the substructure instances form salient groups. Here, the prototype for each substructure class are determined as non-parametric embeddings for better generalization ability [35, 36]. Specifically, the prototype vector $\mathbf{p}_{c}$ is the averaged embedding of the $c$th substructure class. It is updated in the momentum style using the batched substructure embeddings $\tilde{\mathbf{p}}_{c}$:


(9) \begin{align*} & \kern-.5pc \tilde{\mathbf{p}}_{c}=\frac{1}{\vert N(c) \vert}\sum_{k\in N(c)}{\mathbf{z}_{k}}, \end{align*}

(10) \begin{align*} & \mathbf{p}_{c}=\mu \mathbf{p}_{c}+(1-\mu)\tilde{\mathbf{p}}_{c}. \end{align*}

where $N(c)$ is the set of substructure indices that have the highest probability to be assigned to prototype class $c$ in an entire batch of molecules. Since substructures are composed of nodes sharing the same class index, $N(c)$ can be derived from the assignment matrix $\mathbf{S}$. $\tilde{\mathbf{p}}_{c}$ is the average of batched substructure embeddings in class $c$, and $\mu $ denotes the momentum coefficient. In other words, the global estimation of the $\mathbf{p}_{c}$ is updated by its local version $\tilde{\mathbf{p}}_{c}$ in each mini-batch incrementally.

To promote clustering, prototypes and substructures of the same class are viewed as positive pairs, and the rest are viewed as negative pairs. The relation between a prototype and a substructure is measured with cosine similarity:


(11) \begin{align*}& \text{s}(\mathbf{u}, \mathbf{v})=\frac{\mathbf{u}^\top \mathbf{v}}{||\mathbf{u}||\cdot||\mathbf{v}||},\end{align*}

where $\mathbf{u}, \mathbf{v}$ are vector embeddings. We use $\mathbf{p}_{c_{i}}$ to denote the prototype assigned to the $i$th substructure, where $c_{i}$ is the class index with the highest probability assigned by $\mathbf{s}_{i}$ in Equation (3). Then, the prototypical contrastive clustering objective is formulated as follows:


(12) \begin{align*}& \mathcal{L}_{p}=-{\text{log}\frac{\text{exp}\left(\text{s}\left(\mathbf{z}_{i},\mathbf{p}_{c_{i}}\right)/\tau_{2}\right)}{\text{exp}\left(\text{s}\left(\mathbf{z}_{i},\mathbf{p}_{c_{i}}\right)/\tau_{2}\right)+\sum_{c^{\prime}\neq c_{i}}{\text{exp}\left(\text{s}\left(\mathbf{z}_{i},\mathbf{p}_{c^{\prime}}\right)/\tau_{2}\right)}}},\end{align*}

where $\tau _{2}$ is the temperature hyper-parameter. Intuitively, minimizing $\mathcal{L}_{p}$ pushes each transformed substructure embedding ${\mathbf{z}}_{i}$ towards its assigned class prototype $\mathbf{p}_{c_{i}}$ and away from other prototypes.

By clustering substructures of the same class under the guidance of propotypes, the GNN encoder and partitioner coordinately generate high-quality fragmentations. The resultant substructures can thus satisfy the constraints on both topological similarity and attribute consistency.

**Intra-class compactness optimization.** Equation (12) mainly motivates the distinction between substructures and other class prototypes, i.e. inter-class discrimination. Meanwhile, the intra-class similarities shouled be considered. Therefore, we further encourage the compatness within a class by the following loss function [36]:


(13) \begin{align*}& \mathcal{L}_{d}=\left(1-\text{s}\left(\mathbf{z}_{i},\mathbf{p}_{c_{i}}\right)\right)^{2}.\end{align*}

Equation (13) directly minimizes the distance between substructure embeddings and their assigned prototypes. Through this objective, substructures within the same class can have better attribute-based consistency.

To sum up, the clustering objective is formed as follows:


(14) \begin{align*}& \mathcal{L}_{clu} = \mathcal{L}_{p} + \beta \cdot \mathcal{L}_{d}.\end{align*}

Considering the connectivity constraint (7), the overall self-supervised training objective is formulated as follows:


(15) \begin{align*}& \mathcal{L}_{ss} = \gamma \cdot \mathcal{L}_{clu} + (1 - \gamma) \cdot \mathcal{L}_{con}.\end{align*}

where $\beta $ and $\gamma $ are weights of objectives.

After pre-training, POSIT can adaptively identify substructure instances from the input molecules in an unsupervised manner. If new molecular graphs come in, the pre-trained network can be applied conveniently to obtain meaningful substructures, which can then be leveraged to empower molecular representations.

### Fine-tuning stage

The second stage of POSIT is a fine-tuning step. It further optimized the pre-trained network using labelled MPP data, where a cross-scale attention mechanism is introduced to extract informative molecular features for the predictive tasks.

#### Cross-scale attention

Substructures not only provide more contextualized information than individual nodes, but are also at a finer granularity compared with the whole molecule. Therefore, they are supposed to offer rich topological and attribute information. To this end, a cross-scale attention mechanism is devised to explicitly capture the interaction between the substructure-level (local) representations and the graph-level (global) representations to generate informative molecular features.

Globally, a pooling function is operated on the node embeddings of the molecular graph:


(16) \begin{align*}& \mathbf{g} = \text{Readout}(\{\mathbf{h}_{i}, \forall i \in V\}),\end{align*}

where $\mathbf{g} \in \mathbb{R}^{d}$ is the global representation of graph $G$, $V$ is the node set of $G$, and $\text{Readout}(\cdot )$ is a pooling function that compact all nodes in $G$ into a single vector.

Locally, substructure representations are utilized as processing units. Then, the global and local representations are bridged via the cross-scale attention mechanism, which is formed as follows:


(17) \begin{align*} & \theta_{i}^{m} = \frac{\exp ( \sigma ( \mathbf{a}^\top [\mathbf{W}_{m} \mathbf{g} \big\vert\big\vert \mathbf{W}_{m} \mathbf{z}_{i}] ) )}{\sum_{j=1}^{K}{\exp \left( \sigma \left( \mathbf{a}^\top [\mathbf{W}_{m} \mathbf{g} \big\vert\big\vert \mathbf{W}_{m} \mathbf{z}_{j}] \right) \right)}}, \end{align*}

(18) \begin{align*} & \mathbf{g}^{\prime} = \big\vert\big\vert_{m=1}^{M} \sigma \left( \sum_{i=1}^{K}{\theta_{i}^{m} \mathbf{z}_{i}} \right), \end{align*}

where $\mathbf{z}$ is the substructure embedding, $\mathbf{a} \in \mathbb{R}^{2d}$ and $\mathbf{W}_{m} \in \mathbb{R}^{d \times d}$ are trainable parameters to transform the embedding to a scalar, $\vert \vert $ denotes the concatenate operator, $\sigma (\cdot )$ is the activation function, and $M$ is the head count. In this process, substructures serve as fundamental building blocks of the molecule. They are combined linearly, with each substructure weighted according to its attention coefficient relative to the global molecular representation $\mathbf{g}$.

#### Supervised loss function

Given a molecular graph $G$, we combine its summarized embedding $\mathbf{g}$ and the linear combination of the substructure embeddings $\mathbf{g}^{\prime}$ as the final molecular representation:


(19) \begin{align*}& \mathbf{g}_{G} = \left[ \mathbf{g} \vert\vert \mathbf{g^{\prime}} \right].\end{align*}

For binary classification, the representation is fed into another MLP to generate the prediction $\widehat{y} \in \mathbb{R}^{2}$:


(20) \begin{align*}& \widehat{y} = \text{Softmax}\left( \text{MLP}\left( \mathbf{g}_{G} \right) \right).\end{align*}

Considering the potential class imbalance, we adopted the focal loss to tackle the problem [37]. The formula is


(21) \begin{align*} {p_{t}}=\left\{\begin{array}{@{}ll} \widehat{y} &\text{if}\ {y=1} \\ 1 - \widehat{y} &\text{otherwise} \end{array}\right. \end{align*}

(22) \begin{align*} & \mathcal{L}_{sup} = -\sum_{i=1}^{N}{\alpha_{t} \left( 1 - p_{t}^{i} \right)^{\gamma_{t}} \text{log} \left( p_{t}^{i} \right)} \end{align*}

where $y$ is the ground-truth label, $\widehat{y}\in \mathbb{R}$ is the predicted value, and $N$ is the size of dataset. $\alpha _{t}$ is set to 0.25, and $\gamma _{t}$ is set to the proportion of negative samples in experiments.

For regression tasks, the final prediction $\widehat{y}$ is first produced by MLP. Then, MSE loss is adopted as the loss function:


(23) \begin{align*}& \mathcal{L}_{sup} = \frac{1}{N} \sum_{i=1}^{N}{\left( \widehat{y} - y \right)^{2}}.\end{align*}

The overall loss function, including the self-supervised objective and the supervised objective, is formed as follows:


(24) \begin{align*}& \mathcal{L}_{o} = \lambda \cdot \mathcal{L}_{sup} + (1 - \lambda) \cdot \mathcal{L}_{ss},\end{align*}

where $\lambda $ is a hyper-parameter for the trade-off between the supervised loss term and the self-supervised loss term.

## Experiments and results

In this section, we report an extensive set of experimental results including the comparisons with baselines, ablation studies, and visual analysis to demonstrate the effectiveness and substructure identification capability of POSIT.

### Datasets

We mainly consider MPP tasks, including classification and regression. For the pre-training stage, HIV is used as unlabeled pre-training data. It is a molecular dataset originated from MoleculeNet [38], which contains 41 127 molecules in total(Although this dataset is relatively small, it already demonstrates encouraging performance when used as the pre-training dataset for POSIT. We will investigate larger pre-training dataset in our future studies.). The performance of our framework on downstream MPP tasks is evaluated on 10 commonly used datasets from MoleculeNet with labels. These datasets cover a wide range of molecular properties, including physical chemistry, physiology, and biophysics. Among the 10 datasets, 7 are classification tasks and 3 are regression tasks. The statistical information of the datasets is summarized in Table 1, and their detailed descriptions are listed in Supplementary Table S3.

**Table 1:** The statistical information of datasets

**Category**	**Datasets**	**Task type**	#**Molecules**	#**Tasks**	#**Avg nodes**	**Split**	**Metric**
Biophysics	BACE	Classification	1513	1	34.1	Scaffold	ROC-AUC
	HIV	Classification	41 127	1	25.5	Scaffold	ROC-AUC
Physiology	BBBP	Classification	2050	1	23.9	Scaffold	ROC-AUC
	Tox21	Classification	7831	12	18.6	Random	ROC-AUC
	ToxCast	Classification	8597	617	18.7	Random	ROC-AUC
	ClinTox	Classification	1484	2	26.1	Random	ROC-AUC
	SIDER	Classification	1427	27	33.6	Random	ROC-AUC
Physical Chemistry	ESOL	Regression	1128	1	13.3	Random	RMSE
	FreeSolv	Regression	642	1	8.7	Random	RMSE
	Lipophilicity	Regression	4200	1	27.0	Random	RMSE

### Data preprocessing

The molecular data were initially obtained as SMILES strings and then transformed into graph structures using Rdkit [39]. In the first stage, the HIV dataset is used for pre-training. In the fine-tuning stage, all datasets were split into training, validation, and testing subsets with a ratio of 8:1:1. For fair comparisons, we adopted the same data splitting strategy as previous works in terms of random and scaffold splitting [18, 38], which are listed in Table 1. Compared to random splitting, scaffold splitting typically generates datasets that are more challenging for predictive models. Following MoleculeNet [38], the ROC-AUC metric is used for evaluating the performance of the classification tasks, where a higher score means a more accurate prediction. For regression tasks, the Root Mean Squared Error (RMSE) is used as the metric, where a lower score indicates a better result.

### Baseline models

To thoroughly validate our framework, we compare its performance with 8 advanced baseline models. All these baseline models follow the same data preprocessing strategy. The baselines are mainly divided into two categories:

**MPP-oriented GNN variants.** These baselines are introduced to compare the performance of POSIT with models using domain knowledge in chemistry. Among them, attentive FP is a GNN variant based on a multi-level attention mechanism designed for MPP tasks [13]. D-MPNN updates relations between chemical bonds during message passing phases to explicitly include bond information [12]. TrimNet employs a novel triplet message mechanism to calculate messages from atom-bond-atom information for molecular representation [40]. HRGCN+ combines molecular graphs and molecular descriptors as inputs to the GNN model [41]. FP-GNN leverages molecular graphs and fingerprints simultaneously for MPP tasks [14].

**Models with molecular fragmentation rules.** These baselines are introduced to compare the effectiveness of adaptive substructure mining with those methods using pre-defined fragmentation rules. Among them, FraGAT defines all acyclic single bonds as breakable bonds, and randomly chooses a breakable bond to partition the molecule into two fragments [17]. This form of fragmentation is efficient and easy to process. Differently, HiGNN segments a molecule based on the BRICS algorithm, which is a decomposition algorithm based on biochemical domain knowledge [18]. It uses 16 cleavage rules that are pre-defined to decompose the molecules. This approach can obtain an indefinite number of substructures from various molecules.

Besides the GNN-based models and those methods using pre-defined fragmentation rules, we include XGBoost [42, 43], an advanced machine learning algorithm that is a commonly used baseline in MPP tasks.

The input atomic and bond features shared by baseline models are consistent with the one introduced by Xiong *et al*. and Zhu *et al.* [13, 18]. These features are detailed in Supplementary Table S1.

### Experimental setting

**Parameter setup.** In the pre-training stage, we used Attentive FP with edge attributes as the GNN encoder [13]. The model was pre-trained for 100 epochs with the Adam optimizer. During pre-training, the learning rate was adjusted by a consine-based scheduler. After that, the model was fine-tuned for 200 epochs with early stopping. The selection of prototype count $K$ is described in the following Sensitivity Analysis subsection. The trade-off coefficient between losses were set to $\alpha =1$, $\beta =1$, and $\gamma =0.5$, respectively. Other hyper-parameters were optimized by the validation set, and their selection range is detailed in Supplementary Table S4. We performed five independent runs for each dataset with different random seeds. Then, the mean value and standard deviation of their metrics are reported.

**Experimental environment.** Codes for experiments were implemented in the Python. In particular, Pytorch and Pytorch Geometric are the primary third-party libraries we utilized for implementing POSIT. All experiments were carried out using a single 2080Ti GPU.

### Performance validation

To verify the performance of our framework, extensive experiments are conducted on 10 biochemical datasets, covering classification and regression tasks. We compare the performance with MPP-specific models, fragmentation rule-based models and XGBoost. The results of Attentive FP, HRGCN+, and XGBoost were collected from Cai *et al*. [14]. Other results were collected from original studies, respectively. The performance comparisons of POSIT are demonstrated in Table 2.

**Table 2:** Performance comparisons with baseline models

**Dataset**	**Attentive FP**	**D-MPNN**	**HRGCN+**	**TrimNet**	**FP-GNN**	**FraGAT**	**HiGNN**	**XGBoost**	**POSIT**
BACE	0.846	0.857	–	0.878	0.860	0.801	0.882	–	**0.900$\pm $0.028**
Tox21	0.852	0.854	0.848	0.860	0.815	0.843	0.856	0.836	**0.861$\pm $0.017**
ToxCast	0.794	0.764	0.793	0.777	–	0.714	0.781	0.774	**0.796$\pm $0.017**
BBBP	0.909	0.886	–	0.850	0.916	0.923	0.929	–	**0.938$\pm $0.024**
Clintox	0.904	0.897	0.899	0.948	0.840	**0.964**	0.930	0.911	0.845$\pm $0.087
SIDER	0.623	0.658	0.641	0.657	0.661	0.618	0.651	0.642	**0.662$\pm $0.037**
HIV	0.757	0.794	–	0.804	**0.824**	0.761	0.802	–	0.782$\pm $0.025
ESOL	0.587	0.587	0.563	–	0.675	0.536	0.645	0.582	**0.524$\pm $0.013**
FreeSolv	1.091	1.009	0.926	–	**0.905**	1.020	0.915	1.025	1.074$\pm $0.035
Lipophilicity	**0.549**	0.563	0.603	–	0.625	0.651	**0.549**	0.574	0.609$\pm $0.030

Note: the best performance on each dataset is shown in bold. ‘-’ means the results were not reported in the original studies.

As shown in Table 2, POSIT outperforms eight competing baselines in five out of seven datasets for classification tasks. For three regression datasets, it achieves the best performance in ESOL and exhibits competitive performance in Lipophilicity. All these observations indicate that POSIT is capable of effectively predicting a wide range of molecular properties.

The performance are less satisfactory in the FreeSolv, Clintox, and HIV datasets. We notice that FreeSolv has very small data sizes according to Table 1, making it challenging to adapt the pre-trained model to downstream domains. Meanwhile, the average node count of molecules in FreeSolv is the smallest among all datasets. Thus, the substructure classes may be simple and limited, making it difficult to benefit from diverse substructural features. Besides, the distribution of data labels in HIV are extremely unbalanced, which may hinder model’s generalization ability. As for Clintox, it has both the traits of small data size and highly unbalanced data distribution, causing suboptimal results.

### Ablation studies

To further explore the effectiveness of the key components of POSIT, several variants of POSIT have been designed. These variants focus on evaluating the effectiveness of the connectivity objective, the constrastive clustering objective, and the cross-scale attention mechanism:

- **POSIT without connectivity constraint** (w/o Con). This variant removes the modularity and orthogonality-based objectives, which are used to encourage internal connectivity within substructures.
- **POSIT without prototypical contrastive clustering** (w/o Clu). This variant removes the substructure clustering module based on prototype learning. Therefore, substructure identification only relies on the connectivity objective.
- **POSIT without corss-scale attention** (w/o Att). This variant reserves the complete pre-training architecture but removes the cross-scale attention mechanism during fine-tuning. Only the global graph embedding $\mathbf{g}$ is used for prediction.

**Figure 3:**
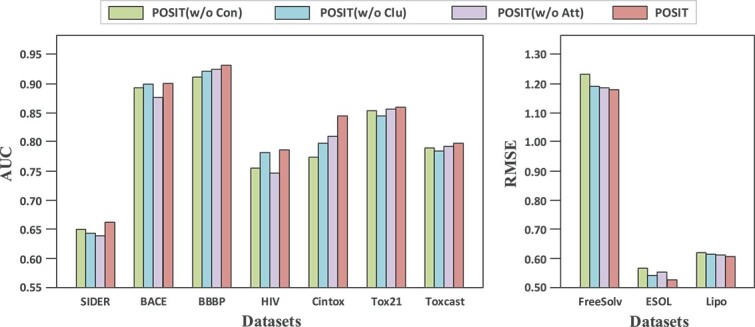
Results of the ablation experiments. **Left:** The results of classification tasks. ROC-AUC is used as the metric. **Right:** The results of regression tasks, using RMSE as the metric.

We have conducted the ablation studies on all datasets adhering to the above experimental setups. The results are demonstrated in Fig. 3.

**Figure 4:**
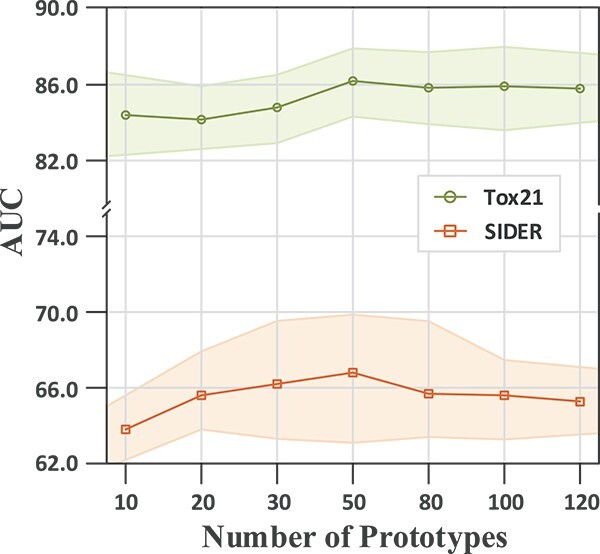
Impact of the number of prototypes K on performance. The shaded area represents the standard deviation.

**Impact of the two pre-training components.** The pre-training consists of two components: connectivity optimization and prototypical contrastive clustering. In Fig. 3, it is evident that POSIT performs better than POSIT w/o Con on all classification and regression datasets, indicating the contribution of connectivity constraints. Performance degradation up to 4% was observed on all datasets. In addition, without the connectivity objective, distant atoms may be partitioned into the same substructure, violating chemical priors. Likewise, an average degradation of 2% is observed on most datasets except for BACE and HIV when using POSIT w/o Clu. The above results indicate that prototypical contrastive clustering leads to more effective substructure identification. Furthermore, the performance rank of POSIT is the most stable on all datasets compared to the variants. Therefore, both components are indispensable for the pre-training stage.

**Impact of the cross-scale attention.** It is observed that POSIT outperforms POSIT w/o Att on all datasets. When removing the cross-scale attention, the performance drops by 3% and 5% on the BACE and HIV datasets, respectively, and an average of 1.5% on other datasets. As a result, it can be inferred that capturing substructural information and fusing hierarchical graph representations can effectively contribute to prediction performance.

### Visualization analysis

In this subsection, we describe qualitative analysis of POSIT through visualization to validate its capability for substructure identification. Concretely, we visualize the distribution of substructures and corresponding prototypes, as well as the identified substructures in each molecule. Additionally, the distributions of substructure counts in each class on different datasets are visualized in Supplementary Fig. S2.

**Figure 5:**
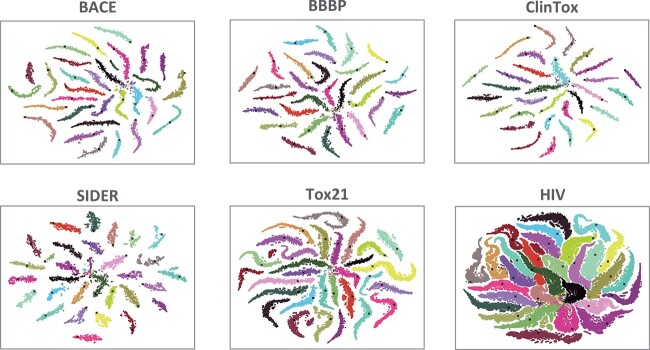
T-SNE visualization of substructure and propotype distribution on six datasets. The prototype count $K$ is set to 30. Each dot represents a substructure, and **$\times $** represents a prototype. Substructures identified as the same class share the same color.

**Distribution of substructures.** Figure 5 visualizes the distribution of structures and prototypes on six datasets after pre-training and fine-tuning. T-SNE is utilized to reduce the dimension of embeddings for visualization [44]. The visualization results of other datasets are demonstrated in Supplementary Fig. 1. In pre-training, the number of prototypes is set to 30 for visual clarity. It can be clearly observed that, around the prototypes, semantically similar substructures will gather closely and form clusters. Meanwhile, there are clear boundaries between different substructure classes. Moreover, there are similar numbers of substructures in different clusters, showing that the orthogonality objective avoids degenerate solutions. Thus, the pre-training stage can effectively identify meaningful substructures in datasets.

**Figure 6:**
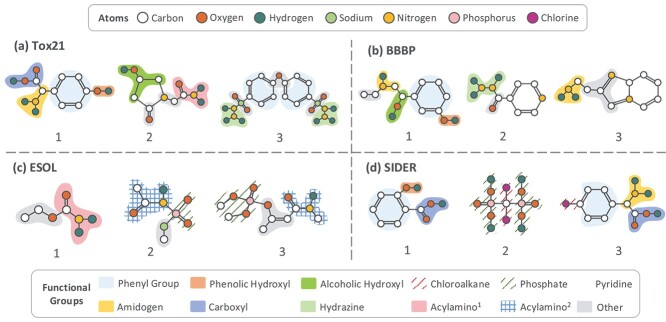
Visualization of the substructures identified. Atoms in a molecule with the same background form a substructure instance. Substructures belonging to the same class across datasets are marked. Substructures that do not match the chemical prior are also marked (which are typically small subgraphs).

**Identification of substructures.** In Fig. 6, we selected three molecules from each of the four datasets and visualized the substructure instances identified in them through the pre-trained network. Specifically, the substructure assignment of node $i$ is determined by the highest probability in $\mathbf{s}_{i}$. Substructures of different classes are marked with distinct background styles (colors and filling types), while those of the same class have identical styles.

It can be observed that nodes in the same substructure are closely connected, and most identified substructures are consistent with chemical priors. For instance, three substructures are identified in the molecule of Fig. 6(d)(1), which correspond to the phenyl group, phenolic hydroxyl group, and carboxyl group, respectively. Besides, substructures marked with the same color share consistent attributes and topology across molecules, which can represent the semantics of a certain functional group. For instance, the carboxyl groups in Fig. 6(a)(1), (d)(1), and (d)(3) are identified as the same class. Also, multiple identical substructures within a molecule are assigned to the same class, and they can also be clearly identified. For instance, in Fig. 6(a)(3), two symmetric hdrazine groups are identified in one molecule. Moreover, some substructures with the same attributes but different structural contexts are distinguished. For instance, the -OH group in Fig. 6(a)(1) and the -OH group in Fig. 6(b(1)) but are identified as different classes, where one represents an alcoholic hydroxyl group and the other represents a phenolic hydroxyl group.

Despite the above advantages, several suboptimal cases are also observed. First, some unrelated atoms are partitioned into certain substructure classes. For instance, although the alcoholic hydroxyl group in Fig. 6(a)(2) is identified, it contains extra carbon atoms than the same group in Fig. 6(b)(1). Additionally, rings in the molecules may be broken. For instance, one of the pyridines in Fig. 6(b)(3) is not clearly identified. However, the probabilistic nature of the partition mitigates such undesired partitions. Different arrangements of the same atoms may also lead to different identifications. For instance, the acylamino groups in Fig. 6(c)(1) and Fig. 6(c)(2) are identified as two different classes in POSIT. There are also substructures that cannot be aligned to known functional groups, such as those marked by gray in Fig. 6(a)(2) and (a)(3).

Overall, despite the suboptimal cases, the formation of numerous substructure classes and their prototypes is satisfactory in general, which corresponds to chemically meaningful functional groups. This claidates the advanced substructure identification capability of the proposed method.

### Sensitivity analysis

In this part, we analyzed the impact of the pre-defined number of prototypes $K$. Figure 4 illustrates the performance of different selections of $K$ ranging from 10 to 120 on SIDER and Tox21. As shown in both datasets, when the initial $K$ is small, performance increases as $K$ increases. When $K$ reaches around 50, performance reaches its peak and then decreases slightly. The performance does not fluctuate violently as $K$ changes. Overall, the performance of POSIT is relatively robust against the choice of the hyper-parameter $K$.

### Conclusion

In this paper, we introduced POSIT, a novel self-supervised approach designed for MPP tasks. The key innovation of POSIT lies in (1) its ability to adaptively identify substructures from molecules; (2) its ability to explicitly incorporate substructure-level information to enhance molecular representations. During pre-training, the connectivity constraint and the prototypical contrastive clustering objective together generate meaningful substructures. In fine-tuning, the cross-scale attention mechanism is leveraged to integrate the substructure-level information to graph-level representations. On this basis, POSIT allows for effective MPP prediction.

We provided a detailed analysis of the performance and capabilities of POSIT. The results of extensive experiments demonstrated POSIT’s effectiveness on MPP tasks. The visualization analysis further validates the advanced capability of POSIT in identifying substructures and aligning them with chemical priors.

Despite the promising results of POSIT, there are still directions for further improvement. For example, real-world molecules are 3D in nature, so incorporating this information in the pre-training will be useful to enhance its effectiveness.

**Key Points**

- We introduce the Prototype-based cOntrastive Substructure IdentificaTion (POSIT) framework, a self-supervised learning approach designed to autonomously discover substructural prototypes across molecular graphs. This innovation allows for the adaptive identification of meaningful substructures to enhance MPP tasks without manual rule definition.
- POSIT employs a two-stage learning process consisting of pre-training and fine-tuning. During pre-training, a graph encoder and partitioner work in tandem to identify substructures, emphasizing connectivity and attribute-based similarity. The fine-tuning stage integrates substructure-level information through a cross-scale attention mechanism, enhancing molecular representations to improve prediction performance.
- Extensive experiments on various real-world datasets demonstrate POSIT’s effectiveness in both classification and regression MPP tasks. The results highlight POSIT’s superior performance compared to multiple baseline models, validating its capability for accurate molecular property prediction.

## Supplementary Material

Revised_Supplementary_Material_bbae565

## Data Availability

Datasets and source codes described in this paper are available at https://github.com/VRPharmer/POSIT.
