# Supplementary material for: Prototype-based contrastive substructure identification for molecular property prediction
Source: Brief Bioinform. 2024 Nov 4;25(6):bbae565. doi: 10.1093/bib/bbae565 (PMC11533112; doi:10.1093/bib/bbae565)
Supplement: Revised_Supplementary_Material_bbae565 [file revised_supplementary_material_bbae565.docx]

**Supplementary Material**

**Gaoqi He^1^, Shun Liu^1^, Zhuoran Liu^1^, Kai Zhang,^1,2,*^, and Honglin Li^2,3,*^**

^1^School of Computer Science and Technology, East China Normal University, Shanghai, China, 200062

^2^Shanghai Key Laboratory of New Drug Design, East China University of Science and Technology, Shanghai, China, 200237

^3^Department of Computer Science and Engineering, East China University of Science and Technology, Shanghai, China, 200237

* To whom correspondence should be addressed.

Contents

**Table S1.** Atomic features used in POSIT

**Table S2.** Bond features used in POSIT

**Table S3.** Descriptions of the datasets used in POSIT

**Table S4.** The candidate hyper-parameter set for optimization

**Table S5.** The prediction performance comparison of POSIT with the random fragmentation strategy

**Table S6**. The prediction performance comparison of POSIT with commonly used machine learning models

**Table S7**. The prediction performance comparison of POSIT with fine-tuning and POSIT with frozen pre-trained parameters

**Table S8**. The prediction performance comparison of POSIT with the model that replace substructure features with fingerprint features

**Table S9**. The prediction performance comparison between POSIT and advanced ADMET models

Figure S1. The distribution of substructures and prototypes of ESOL, FreeSolv, Lipo and Toxcast

Figure S2. The count of substructures in each class of BACE(above) and BBBP (below) after pre-training

Reference

**Table S1.** Atomic features used in POSIT

| **Feature** | **Size** | **Description** |
| --- | --- | --- |
| atom symbol | 16 | [B, C, N, O, F, Si, P, S, Cl, As, Se, Br, …] (one-hot vector) |
| degree | 7 | number of covalent bonds, [0,1,2,3,4,5, other] (one-hot vector) |
| formal charge | 1 | number of electrical charges |
| radical electrons | 1 | number of radical electrons |
| hybridization | 6 | [sp, sp^2^, sp^3^, sp^3^d, sp^3^d^2^, other] (one-hot vector) |
| aromaticity | 1 | if the atom is part of an aromatic system: 1 else: 0 |
| hydrogens | 5 | number of connected hydrogens [0,1,2,3,4] (one-hot vector) |
| chirality | 1 | if the atom is chiral center: 1 else: 0 |
| chirality type | 2 | [R, S] (one-hot vector) |
| donor | 1 | if the atom is hydrogen bond donor: 1 else: 0 |
| acceptor | 1 | if the atom is hydrogen bond acceptor: 1 else: 0 |
| basic | 1 | if the atom is part of basic: 1 else: 0 |
| acid | 1 | if the atom is part of acid: 1 else: 0 |
| halogen | 1 | if the atom is halogen: 1 else: 0 |
| scaffold | 1 | if the atom is part of Murcko scaffold: 1 else: 0 |

There are 46 bits of atomic features in total. The top 39 bits of atomic features are introduced by Attentive FP [1]. Additional “other” option is added to the number of covalent bonds as HiGNN [2] suggested. Pharmacophore features (donor, acceptor, basic, acid, halogen) and scaffold information are also adopted by HiGNN.

**Table S2.** Bond features used in POSIT

| **Feature** | **Size** | **Description** |
| --- | --- | --- |
| bond type | 4 | [single, double, triple, aromatic] (one-hot vector) |
| conjugation | 1 | if the bond is conjugated: 1 else: 0 |
| inside ring | 1 | if the bond is inside of a ring: 1 else: 0 |
| stereo | 4 | [StereoNone, StereoAny, StereoZ, StereoE] (one-hot vector) |

There are 10 bits of bond features in total. The setup of bond features is followed by Attentive FP [1].

**Table S3.** Descriptions of the datasets used in STEM

| **Dataset** | **Category** | **Description** |
| --- | --- | --- |
| BACE | Biophysics | Binary classification dataset that describes drug-target binding affinity for a set of inhibitors of human β-secretase |
| HIV | Biophysics | Binary classification dataset for testing the ability to inhibit HIV replication |
| BBBP | Physiology | Binary classification dataset for barrier permeability of drugs, which is critical for central nervous system |
| Tox21 | Physiology | Multi-label classification dataset for different targets including nuclear receptors and stress response pathways |
| ToxCast | Physiology | Multi-label dataset for toxicology data for a large library of compounds based on high-throughput screening. |
| ClinTox | Physiology | 2-label classification dataset for predicting toxicity in clinical trials and the FDA approval status |
| SIDER | Physiology | Multi-label classification dataset for predicting whether the drug is in the side effect group. |
| ESOL | Physical Chemistry | Regression dataset for predicting water solubility (log solubility in mols per liter) of compounds |
| FreeSolv | Physical Chemistry | Regression dataset for predicting the hydration free energy of molecules in water |
| Lipophilicity | Physical Chemistry | Regression dataset for predicting the octanol/water distribution coefficient (logD at pH7.4) of compounds |

The descriptions of datasets are summarized from MoleculeNet [3].

**Table S4.** The candidate hyper-parameter set for optimization

| **Parameters** | **Search Space** |
| --- | --- |
| hidden dimension of features | {64, 128, 256} |
| layers of the GNN encoder | {2, 3, 4} |
| batch size | {64, 128, 256, 512} |
| dropout rate | {0.1, 0.2, 0.3, 0.4} |
| learning rate | {1e-4, 5e-4,1e-3,5e-3} |
| weight decay | {1e-4, 5e-5, 1e-5} |
| $\tau_{1}$ (temperature of softmax) | {0.1, 0.5, 1} |
| $\tau_{2}$ (temperature of contrastive loss) | {0.1, 0.5, 1} |
| $\mu$ (momentum coefficient) | {0.95, 0.99, 0.999} |
| $K$ (number of prototypes) | {10, 20, 30, 50, 80, 100, 120} |
| $m$ (number of heads) | {1, 2, 4, 8} |
| $\gamma$ (weights of loss terms) | {0.1, 0.3, 0.5, 0.7} |

The hyper-parameters are optimized through the random split strategy on the validation set.

**Table S5.** The prediction performance comparison of POSIT with the random fragmentation strategy

| **Dataset** | **Metric** | **POSIT with Random Fragmentation** | **POSIT** |
| --- | --- | --- | --- |
| BACE | ROC-AUC | 0.807±0.064 | **0.900±0.028** |
| HIV | ROC-AUC | 0.718±0.034 | **0.782±0.025** |
| BBBP | ROC-AUC | 0.905±0.037 | **0.938±0.024** |
| Tox21 | ROC-AUC | 0.854±0.019 | **0.861±0.017** |
| ToxCast | ROC-AUC | 0.785±0.018 | **0.796±0.017** |
| ClinTox | ROC-AUC | **0.849±0.049** | 0.845±0.087 |
| SIDER | ROC-AUC | 0.653±0.021 | **0.662±0.037** |
| ESOL | RMSE | 0.540±0.080 | **0.524±0.013** |
| FreeSolv | RMSE | 1.886±0.062 | **1.074±0.035** |
| Lipophilicity | RMSE | 0.663±0.023 | **0.609±0.030** |

To demonstrate the necessity of proper fragmentations for MPP tasks, we have applied the random fragmentation strategy to POSIT, and compared its performance with the standard POSIT. In specific, we follow Mesquita1 et al. [4] to replace the assignment matrix S in Equation (4) with a normalized random matrix $\tilde{\mathbf{S}}$, where ${\tilde{\mathbf{S}}}_{\mathbf{i},\mathbf{j}}\sim N(0, 1)$, and $N$ denotes the Gaussian distribution. Other experimental settings remain the same as described in the main text.

As shown in Table S5, POSIT with adaptive fragmentation, i.e., the standard POSIT outperforms POSIT with random fragmentation on 9 out of 10 datasets. The improvement of the model is particularly significant on BACE, HIV, and Lipophilicity (over 5%). The only exception is ClinTox, which means that the pre-trained substructure features cannot transfer well to its tasks. Pre-training on a larger scale may improve this situation.

Overall, this comparison POSIT with adaptive fragmentation achieved better performance, proving the effectiveness and necessity of adaptive fragmentation in POSIT.

**Table S6.** The prediction performance comparison of POSIT with commonly used machine learning models

| **Dataset** | **Metric** | **RF** | **SVM** | **LogReg** | **POSIT** |
| --- | --- | --- | --- | --- | --- |
| BACE | ROC-AUC | 0.866±0.003 | 0.865±0.000 | 0.798±0.006 | **0.900±0.028** |
| HIV | ROC-AUC | 0.702±0.004 | 0.774±0.000 | 0.702±0.015 | **0.782±0.025** |
| BBBP | ROC-AUC | 0.708±0.006 | 0.722±0.000 | 0.699±0.004 | **0.938±0.024** |
| Tox21 | ROC-AUC | 0.755±0.004 | 0.801±0.001 | 0.767±0.011 | **0.861±0.017** |
| ToxCast | ROC-AUC | 0.664±0.005 | 0.676±0.002 | 0.610±0.006 | **0.796±0.017** |
| ClinTox | ROC-AUC | 0.715±0.002 | 0.832±0.001 | 0.834±0.002 | **0.845±0.087** |
| SIDER | ROC-AUC | 0.658±0.002 | 0.638±0.017 | 0.601±0.010 | **0.662±0.037** |
| ESOL | RMSE | 1.165±0.013 | 1.229±0.000 | - | **0.524±0.013** |
| FreeSolv | RMSE | 2.016±0.018 | 1.990±0.000 | - | **1.074±0.035** |
| Lipophilicity | RMSE | 0.854±0.004 | 0.777±0.000 | - | **0.609±0.030** |

Here, we have compared the prediction performance of POSIT with 3 models that are used as benchmarks in MoleculeNet [3], including Random Forest (RF), Support Vector Machine (SVM), and Logistic Regression (LogReg). In specific, we tested the performance of MPP tasks with the Random Forest model with 500 trees, the SVR / SVC model with penalty parameter = 1.0, and the Logistic Regression model with penalty parameter = 1.0. All machine learning models used ECFP as initial features of input molecules. Other experimental settings remain the same as described in the main text.

As shown in Table S6, POSIT achieves the best performance on 10 datasets over these 3 machine learning models, demonstrating its effectiveness on MPP tasks. Meanwhile, it can be observed that machine learning models typically have lower standard deviation, showing their stability and robustness towards downstream tasks. If supplemented with appropriate feature selection methods, their performance may further be improved, and their advantages in interpretability can be exerted.

**Table S7.** The prediction performance comparison of POSIT with fine-tuning and POSIT with frozen pre-trained parameters

| **Dataset** | **Metric** | **POSIT (frozen params)** | **POSIT (fine-tuning)** |
| --- | --- | --- | --- |
| BACE | ROC-AUC | 0.844±0.029 | **0.900±0.028** |
| HIV | ROC-AUC | 0.718±0.034 | **0.782±0.025** |
| BBBP | ROC-AUC | 0.881±0.040 | **0.938±0.024** |
| Tox21 | ROC-AUC | 0.802±0.007 | **0.861±0.017** |
| ToxCast | ROC-AUC | 0.719±0.020 | **0.796±0.017** |
| ClinTox | ROC-AUC | 0.692±0.087 | **0.845±0.087** |
| SIDER | ROC-AUC | 0.627±0.030 | **0.662±0.037** |
| ESOL | RMSE | 0.540±0.080 | **0.524±0.013** |
| FreeSolv | RMSE | 1.886±0.602 | **1.074±0.035** |
| Lipophilicity | RMSE | 0.663±0.023 | **0.609±0.030** |

To demonstrate the effectiveness of the fine-tuning process, we have compared the standard fine-tuned POSIT with POSIT with frozen pre-trained parameters. In implementing the latter model, we frozen the pre-trained parameter, only training the MLP-based classifier or regressor based on supervised signals. In this case, the pre-trained model is used as a fixed feature extractor. Other experimental settings remain the same as described in the main text.

As shown in Table S7, we can observe that the standard POSIT with fine-tuning achieves better performance on 10 datasets. Meanwhile, the performance gap is obvious on all classification and regression dataset. These results demonstrate the necessity of fine-tuning the pre-trained parameters of the model to adapt it to downstream tasks. In POSIT, the GNN-based graph encoder used in Equation (1) and the MLP-based node partitioner used in Equation (2) can be fine-tuned.

**Table S8.** The prediction performance comparison of POSIT with the model that replace substructure features with fingerprint features

| **Dataset** | **Metric** | **POSIT with FP** | **POSIT** |
| --- | --- | --- | --- |
| BACE | ROC-AUC | 0.867±0.035 | **0.900±0.028** |
| HIV | ROC-AUC | 0.718±0.034 | **0.782±0.025** |
| BBBP | ROC-AUC | 0.898±0.042 | **0.938±0.024** |
| Tox21 | ROC-AUC | 0.834±0.014 | **0.861±0.017** |
| ToxCast | ROC-AUC | 0.753±0.030 | **0.796±0.017** |
| ClinTox | ROC-AUC | 0.818±0.068 | **0.845±0.087** |
| SIDER | ROC-AUC | 0.621±0.017 | **0.662±0.037** |
| ESOL | RMSE | 0.713±0.056 | **0.524±0.013** |
| FreeSolv | RMSE | 1.987±0.068 | **1.074±0.035** |
| Lipophilicity | RMSE | 0.697±0.026 | **0.609±0.030** |

In this setting, we have replaced the substructure features of POSIT with fingerprint (FP) features to compare their performance. Specifically, we concatenated ECFP (Morgan) fingerprints and MACCS fingerprints as the initial FP features of a molecule. Then, MLP layers are used to encode these features. Finally, we concatenated GNN-encoded features and the FP features to predict task labels. Attentive FP is used as the GNN model, which is the same as used in POSIT. Other experimental settings remain the same as described in the main text.

As shown in Table S8, POSIT with substructure features (the standard POSIT) outperforms POSIT with FP features on all datasets. Moreover, the standard deviation of POSIT with FP is also larger. These results demonstrate that, although molecular fingerprints also encode substructure information to some extent, they are not as effective as the substructure features adaptively mined by POSIT.

**Table S9.** The prediction performance comparison between POSIT and advanced ADMET models

| **Dataset** | **Metric** | **Property** | **ADMET lab** | **ADMET-PrInt** | **POSIT** |
| --- | --- | --- | --- | --- | --- |
| ESOL | RMSE | logS | 0.542 | **-** | **0.524±0.013** |
| Solub | RMSE | logS | - | 0.767±0.013 | **0.704±0.047** |

ADMET researches primarily focus on molecular properties of absorption, distribution, metabolism, and excretion. Some of the datasets used by ADMET studies are consistent with our study. Here, we conducted experiments to compare the prediction performance of POSIT with two advanced ADMET models [5, 6]. For fair comparison, we chose two publicly available datasets and the property shared by POSIT and the ADMET models. The Solub dataset is available at the original study of ADMET-PrInt. In implementation, the prediction results of both ADMET models requires to be requested from online services. Other experimental settings remain the same as described in the main text.

As shown in Table S9, POSIT achieves better performance on both datasets against the ADMET models. In future works, we plan to extend POSIT to include a broader range of molecular properties and datasets. By incorporating additional ADMET-related properties such as metabolism, we may enhance the applicability of POSIT in modern drug discovery pipeline.

**
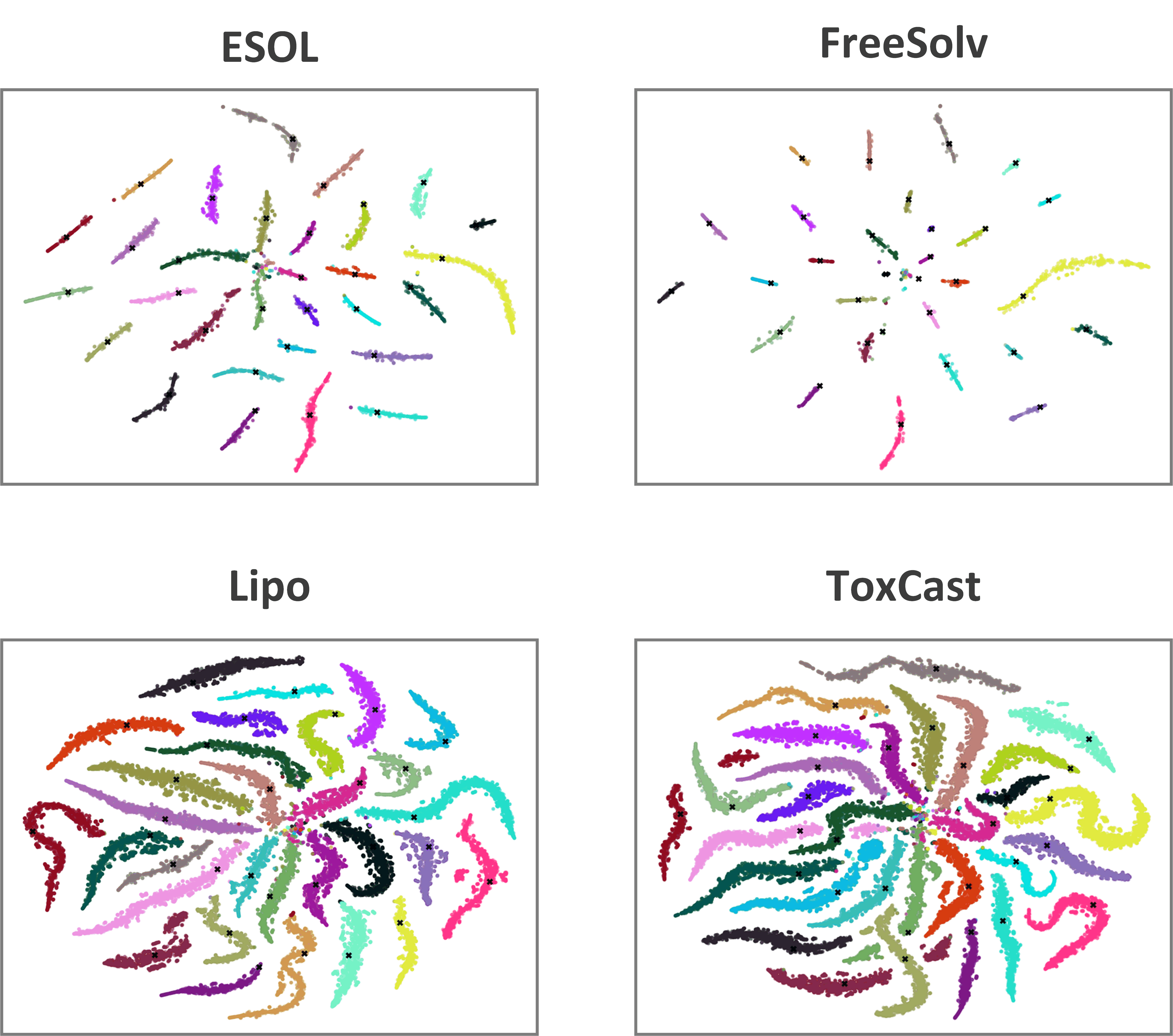
**

**Figure S1.** The distribution of substructures and prototypes of ESOL, FreeSolv, Lipo and Toxcast. The visualization setup is consistent with the one in main text.


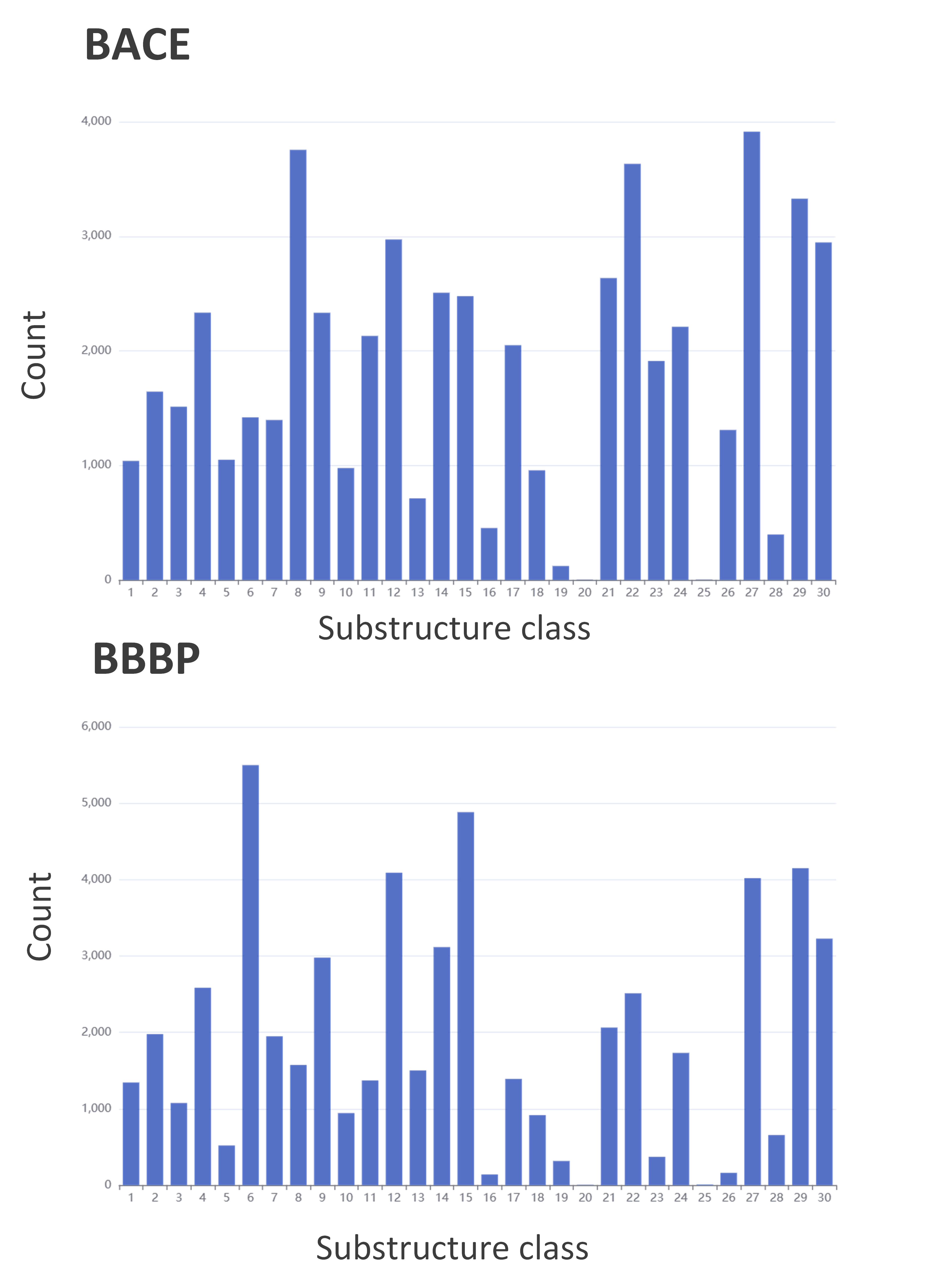


**Figure S2.** The count of substructures in each class of BACE(above) and BBBP (below) after pre-training. $\mathbf{K}$ is set to 30. It is observed that classes are not strictly balanced due to the imbalanced nature of substructure distribution. After pre-training, the count of the same substructure class differs in different datasets, but its proportion to the overall count is similar across datasets.

**Reference**

[1] Zhaoping Xiong, Dingyan Wang, Xiaohong Liu, Feisheng Zhong, Xiaozhe Wan, Xutong Li, Zhaojun Li, Xiaomin Luo, Kaixian Chen, Hualiang Jiang, et al. Pushing the boundaries of molecular representation for drug discovery with the graph attention mechanism. *Journal of medicinal chemistry*, 63(16):8749–8760, 2020.

[2] Weimin Zhu, Yi Zhang, Duancheng Zhao, Jianrong Xu, and Ling Wang. Hignn: A hierarchical informative graph neural network for molecular property prediction equipped with feature-wise attention. *Journal of chemical information and modeling*, 63(1):43–55, 2023.

[3] Zhenqin Wu, Bharath Ramsundar, Evan N Feinberg, Joseph Gomes, Caleb Geniesse, Aneesh S Pappu, Karl Leswing, and Vijay Pande. Moleculenet: a benchmark for molecular machine learning. *Chemical science*, 9(2):513–530, 2018.

[4] Mesquita D, Souza A, Kaski S. Rethinking pooling in graph neural networks. *Advances in Neural Information Processing Systems*, 33: 2220-2231, 2020.

[5] Li Fu, Shaohua Shi, Jiacai Yi, Ningning Wang, Yuanhang He, Zhenxing Wu, Jinfu Peng, et al. ADMETlab 3.0: an updated comprehensive online ADMET prediction platform enhanced with broader coverage, improved performance, API functionality and decision suppor. *Nucleic Acids Research*, gkae236, 2024.

[6] Ewelina Jamrozik, Marek Smieja, and Sabina Prdlewska. ADMET-PrInt: Evaluation of ADMET Properties: Prediction and Interpretation, Journal of Chemical Information and Modeling, 64(5): 1425–1432, 2024.
